# Feedback from lateral occipital cortex to V1/V2 triggers object completion: Evidence from functional magnetic resonance imaging and dynamic causal modeling

**DOI:** 10.1002/hbm.25637

**Published:** 2021-08-21

**Authors:** Siyi Chen, Ralph Weidner, Hang Zeng, Gereon R. Fink, Hermann J. Müller, Markus Conci

**Affiliations:** ^1^ Department of Psychology Ludwig‐Maximilians‐Universität München München Germany; ^2^ Cognitive Neuroscience Institute of Neuroscience and Medicine (INM‐3) Research Center Jülich Jülich Germany; ^3^ Center for Educational Science and Technology Beijing Normal University at Zhuhai Zhuhai China; ^4^ Department of Neurology University Hospital Cologne Cologne University Cologne Germany

**Keywords:** effective connectivity, feedback connections, illusory figure, modal completion, object integration

## Abstract

Illusory figures demonstrate the visual system's ability to integrate disparate parts into coherent wholes. We probed this object integration process by either presenting an integrated diamond shape or a comparable ungrouped configuration that did not render a complete object. Two tasks were used that either required localization of a target dot (relative to the presented configuration) or discrimination of the dot's luminance. The results showed that only when the configuration was task relevant (in the localization task), performance benefited from the presentation of an integrated object. Concurrent functional magnetic resonance imaging was performed and analyzed using dynamic causal modeling to investigate the (causal) relationship between regions that are associated with illusory figure completion. We found object‐specific feedback connections between the lateral occipital cortex (LOC) and early visual cortex (V1/V2). These modulatory connections persisted across task demands and hemispheres. Our results thus provide direct evidence that interactions between mid‐level and early visual processing regions engage in illusory figure perception. These data suggest that LOC first integrates inputs from multiple neurons in lower‐level cortices, generating a global shape representation while more fine‐graded object details are then determined via feedback to early visual areas, independently of the current task demands.

## Graphical abstract

The visual system has a remarkable ability to integrate disparate parts into coherent whole objects, as illustrated, for example, by the perception of illusory figures. This study investigates connectivity patterns associated with illusory figures and provides direct evidence that the formation of completed objects relies on feedback connections from mid‐level to early visual processing regions: LOC first generates a global shape representation while more fine‐graded object details are then determined via (back‐) projections to early visual areas, independently of the current task demands.

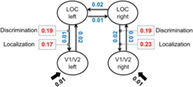

## 1 INTRODUCTION

**FIGURE 1:**
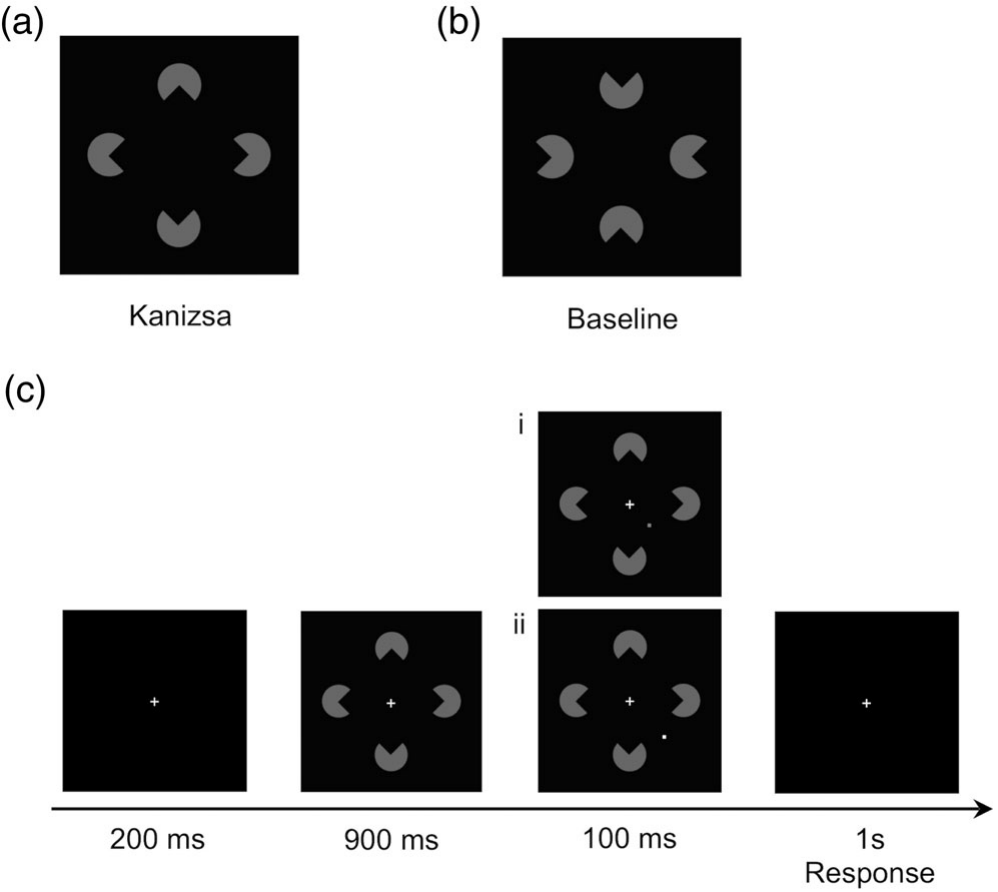
Examples of the stimuli used in the experiment: (a) Kanizsa figure that induces an illusory diamond shape; (b) Baseline configuration presenting comparable pacman items, but without inducing a comparable illusory object. (c) Example trial sequence in the main experiment: following a fixation cross (200 ms), a configuration (either Kanizsa or Baseline) was briefly presented (900 ms), after which a target (dot‐probe) was added and presented for another 100 ms (followed by a 1‐s response interval). In the example, the target was presented near the bottom‐right boundary of the enclosed region. In the luminance discrimination task, observers were instructed to report whether the target was light or dark gray (here, the correct response would be “dark” [i] and “light” [ii]). In the spatial localization task, they were asked to indicate whether the target appeared inside or outside the enclosed illusory region (in the examples, the correct response would be “inside” [i] and “outside” [ii])

Perceiving meaningful visual objects in our cluttered environment requires that the visual system combines disparate parts into coherent wholes, as demonstrated, for example, in Kanizsa‐type illusory figures. For instance, as depicted in Figure 1a, a configuration of four “pacman” elements generates the perception of a diamond‐shaped illusory object (a “Kanizsa” figure) with a surface that appears to be brighter than the background and sharp boundaries that seem to occlude the adjacent circular elements. In contrast, the “Baseline” control configuration (Figure 1b) does not induce object completion processes to the same extent and, hence, no illusory figure emerges, even though it consists of similar inducer elements that likewise present a symmetric pacman arrangement.

Findings from human neuroimaging and neurophysiological studies show that both lower‐ (V1/V2) and higher‐tier visual cortices (particularly the lateral occipital cortex [LOC]) are implicated in the processing of illusory figures (e.g., Bakar, Liu, Conci, Elliott, & Ioannides, 2008; Chen et al., 2020; Ffytche & Zeki, 1996; Hirsch et al., 1995; Kok & de Lange, 2014; Lee & Nguyen, 2001; Maertens & Pollmann, 2007; Mendola, Dale, Fischl, Liu, & Tootell, 1999; Peterhans & von der Heydt, 1989; Ritzl et al., 2003; Seghier et al., 2000; Stanley & Rubin, 2003). For instance, Chen et al. (2020) employed functional magnetic resonance imaging (fMRI) combined with retinotopic mapping to track the neuronal object completion process by presenting different variants of Kanizsa figures that incrementally increased in grouping strength. On each trial, one type of configuration was presented together with a small target dot, and observers were asked to either determine the spatial location of the dot (inside vs. outside of the presented Kanizsa‐type configuration; see also Chen, Glasauer, Müller, & Conci, 2018), or its brightness (light vs. dark gray; see also Weidner & Fink, 2007; Plewan, Weidner, Eickhoff, & Fink, 2012). Of note, the two tasks differed in terms of their attentional requirements: in the spatial localization task, the Kanizsa‐type configuration was directly task relevant; in the brightness discrimination task, in contrast, the brightness of the target dot could be discerned without relating it to the surrounding object configuration. Following previous findings (see references above), the results revealed bilateral LOC and early visual cortex to be both involved in the processing of the illusory figure, with an object‐specific modulation evident in both task conditions, that is, independently of the task's attentional requirements. Moreover, LOC was particularly associated with variations in grouping strength: its activation scaled with the presentation of more versus less complete objects. Together, these findings indicate that integrated objects are generated during early and mid‐level visual processing independently of the current top‐down‐instantiated task set (see also Han, Jiang, Mao, Humphreys, & Gu, 2005). However, the specific interaction scheme across separate regions involved in object completion has not been demonstrated so far. Therefore, the current study aimed to extend this previous work by investigating patterns of effective connectivity between the regions relevant for object integration.

Influential models of object integration in general (Hochstein & Ahissar, 2002; Lamme & Roelfsema, 2000; Roelfsema, 2006) and illusory figure completion, in particular, posit two complementary routes of neuronal communication: a feedforward sweep of information (Ffytche & Zeki, 1996; Grosof, Shapley, & Hawken, 1993; Leventhal, Wang, Schmolesky, & Zhou, 1998; Sheth, Sharma, Rao, & Sur, 1996) and a reverse, recurrent processing architecture (Lee & Nguyen, 2001; Stanley & Rubin, 2003). Pure feedforward processing accounts assume that object completion begins in lower‐tier visual areas, where basic features of the presented stimulus are processed. Perceptual stimulus analysis then proceeds by progressively transferring information to areas higher up in the visual hierarchy, which, in turn, process more complex stimulus attributes (e.g., Ffytche & Zeki, 1996; Grosof et al., 1993; Sheth et al., 1996). In contrast, recurrent processing accounts assume that information is integrated across different levels of the visual hierarchy by a combination of feedforward and feedback connections. On this view, modulations observed in the early visual cortex in response to illusory figures, rather than just reflecting the initial stimulus analysis, might also reflect feedback from higher‐order visual regions (e.g., Foxe, Murray, & Javitt, 2005; Lee & Nguyen, 2001; Mendola et al., 1999). Such feedback connections might serve to process a complete object's finer details. For instance, initial feedforward processing might foster the (relatively crude) segregation of the illusory figure from the background, while feedback connections would subsequently render details about the specific (illusory) contour representation (see also Conci, Groß, Keller, Müller, & Finke, 2018; Nowack et al., 2021; Roelfsema, 2006; Stanley & Rubin, 2003). The purpose of the current study was to test these two alternative hypotheses about the potential connectivity between early visual areas and LOC.

Direct tests of potential interactions between illusory figure‐sensitive regions using methods with relatively high temporal resolution suggest that responses to illusory figure perception in LOC do occur earlier in time than corresponding responses in early visual areas (e.g., Murray et al., 2002; Murray, Foxe, Javitt, & Foxe, 2004; Halgren, Mendola, Chong, & Dale, 2003; Yoshino et al., 2006; Bakar et al., 2008; Shpaner, Molholm, Forde, & Foxe, 2013; Wokke, Vandenbroucke, Scholte, & Lamme, 2013; for a review, see Murray & Herrmann, 2013). Such findings appear to contradict the notion that the early visual cortex initiates a fast‐latency, stimulus‐driven signal to higher‐order visual regions, as hypothesized by pure feedforward processing models. For instance, in an magnetoencephalography (MEG) study, Halgren et al. (2003) showed that a prominent peak of differential activity in response to Kanizsa figures (vs. a comparable, ungrouped configuration) occurs at ~155 ms in the LOC. This figure‐specific modulation then appears to spread back from LOC towards the occipital pole, revealing a later peak in earlier visual areas (V1/V2; see also Yoshino et al., 2006). A comparable (recurrent) sequence of processing between LOC and early visual areas was also found in an assessment of the spatio‐temporal dynamics of brain activity through high‐density electrophysiological recordings combined with an inverse source analysis (Shpaner et al., 2013; see also Knebel & Murray, 2012). Moreover, Wokke et al. (2013) used transcranial magnetic stimulation to disrupt signaling in V1/V2 and LOC at different time points while participants performed an illusory‐figure discrimination task. The results revealed an early disruption of neural signaling in LOC to degrade performance, while disruption of neural signaling over V1/V2 reduced performance particularly during later time points—again supporting models, which assume recurrent interactions between mid‐level and early visual cortex. Thus, while the importance of feedback connections for object completion has been demonstrated using various neurophysiological methods, there has been little direct evidence from neuroimaging studies for the causal interaction between the early visual cortex and LOC in the processing of illusory figures in humans. This is partly owing to the fact that the blood oxygen level‐dependent (BOLD) response in fMRI is slow, precluding conclusions about temporal causality when using conventional methods.

Given this, the principal aim of the current study was to test, for the first time, the effective connectivity between early visual cortex (V1/V2) and LOC in response to a complete illusory figure while varying attentional task demands (through instruction). Dynamic causal modeling (DCM) is a technique that provides a validated estimate of effective connectivity, reflecting the directional coupling between neuronal populations (Friston, Harrison, & Penny, 2003). DCM may thus provide valuable information to complement previous findings that used other techniques in order to decide between possible connection schemes between regions implicated in illusory figure processing. Accordingly, we used DCM to assess the cortical dynamics in time between brain regions in a bilinear fashion (Friston et al., 2003; Penny, Stephan, Mechelli, & Friston, 2004) by combining both neuroimaging and behavioral data for the (fully grouped) Kanizsa figure and the ungrouped (baseline) configurations from our previous study (Chen et al., 2020). Three model variants were tested, all of which included V1/V2 and LOC as representative nodes. This allowed assessment of how connections between these nodes vary as a function of illusory figure completion and task requirements (thus further testing the specific role of attention in illusory figure completion). If V1/V2 is initially involved in constructing a whole object representation, then connectivity should increase in a feedforward manner from V1/V2 to LOC given an illusory figure as perceptual input. Alternatively, if the completion of the illusory figure originates at higher levels in LOC (so that V1/V2 would be involved only subsequently), this would instead support an account of object completion in terms of feedback processing. Finally, the integration of the illusory figure could also be reflected in bidirectional processing.

## 2 MATERIALS AND METHODS

### 2.1 Participants

Twenty‐three right‐handed adults with normal or corrected‐to‐normal visual acuity participated in the fMRI experiment. Three participants were excluded from analysis due to excessive head motion (more than 3 mm of displacement or 3° of rotation in any direction) during scanning or because they committed a relatively high proportion of response errors (exceeding 3 *SD* above mean performance)—thus, leaving the data from 20 participants (11 women, mean age = 27.5 years, *SD* = 6.4) for analysis. All participants were remunerated for their participation and gave informed consent before the experiment. The ethics committee of the Department of Psychology, Ludwig‐Maximilians‐Universität München, approved the experimental procedures. The sample size was sufficient to detect a difference between Kanizsa and Baseline configurations in a repeated‐measures analysis of variance (ANOVA) based on the effect sizes (with f(U) values ranging between 0.8 and 1.4) as derived from previous, similar fMRI studies (e.g., Kok & de Lange, 2014; Maertens, Pollmann, Hanke, Mildner, & Möller, 2008; Mendola et al., 1999), with 85% power and an alpha level of .05. Moreover, the sample size tested in the current study was comparable to (or even larger than) other, recent visual‐attention fMRI studies that also employed similar DCM analyses (e.g., Plewan et al., 2012; Vossel, Mathys, Stephan, & Friston, 2015).

### 2.2 Stimuli

Stimuli were generated with an IBM‐PC compatible computer using Matlab routines and Psychophysics Toolbox extensions (Brainard, 1997; Pelli, 1997) and were presented in light gray (RGB: 103, 103, 103) against a black (RGB: 0, 0, 0) background at the center of a 30‐in shielded LCD monitor mounted outside the scanner on the wall behind the subject's head. The screen was located at a distance of 245 cm from the participant. It was seen via a mirror on top of the head coil. There were two types of experimental stimuli (see Figure 1a,b)^1^: (a) a Kanizsa diamond configuration (Kanizsa), and (b) a control configuration that consisted of four “pacman” inducers that were exactly the same as those in the Kanizsa configuration, but with their indents facing away from the stimulus center. Thus, this baseline configuration depicted a symmetric arrangement but without presenting any object information, for example, an illusory shape. Each pacman inducer subtended a visual angle of 1.5°. The distance from the center of the illusory diamond shape was 2.7° of visual angle. The support ratio (Banton & Levi, 1992), that is, the ratio between the luminance‐defined portion and the completed illusory contour was 0.4, which leads to the impression of a clearly visible illusory figure.

An additional small dot‐probe (9 arc‐min in diameter) served as the target stimulus, which was randomly presented in light (RGB: 220, 220, 220) or dark (RGB: 78, 78, 78) gray close to the illusory edge of a given pacman configuration in the lower left or right display quadrant. The dot‐probe appeared randomly at one of two equidistant locations along the midline perpendicular to the bottom left or right border of the illusory figure (−14 or + 14 arc‐min from the center point of the border). These location parameters were derived from our previous, behavioral study (Chen et al., 2018) and have previously shown to reveal a reliable and substantial difference in performance. This dot‐probe was added to one of the two possible configurations (Kanizsa or Baseline). Note that we probed the lower left and right quadrants of the display because the lower hemifield has been shown to produce a more robust percept of an illusory figure than the upper hemifield (Rubin, Nakayama, & Shapley, 1996).

### 2.3 Procedure and design

To examine whether and how attending to a given to‐be‐grouped configuration impacts object integration, we manipulated the attentional demands using two tasks: a spatial localization and a luminance discrimination task. In the spatial localization task, participants indicated whether the dot probe was located inside or outside of the perceived illusory region enclosed by the inducers. In the luminance discrimination task, participants indicated whether the dot‐probe on the figure was light or dark gray. Participants responded by pressing the left and right button with their left (inside/light) or right (outside/dark) index finger, respectively. The physical stimuli were the same in both the spatial localization and the luminance discrimination task. However, to accurately locate the dot‐probe near the boundary, the presented configuration's contour must be taken into account and thus attended. In contrast, for the luminance discrimination task, the surrounding stimulus configuration was mostly irrelevant. This task could therefore be performed without explicitly attending to the neighboring configuration.

The experiment employed a blocked design: each experimental block (with eight trials each) presented one, fixed stimulus type, with 20 blocks for the Kanizsa configuration and 20 blocks for the Baseline configuration. Within each block, the target dot appeared always on the same side (bottom left or right) of the presented configuration (i.e., there were 10 blocks per Kanizsa/baseline configuration and left/right side)—ensuring that attention could be consistently allocated toward a single, repeating stimulus type and dot location. All the stimuli (and target side) blocks were randomly interleaved but presented separately for each type of task. A semantic cue was presented for 5 s at the start of each task session, informing the participants whether the luminance discrimination task or the spatial localization task had to be performed. A blank screen with a fixation cross was presented for 5 s at the start of each task session and the end of each block as well as the end of the whole experiment. The two task sessions were presented in a randomized order, separated by periods that presented the fixation cross or the task instructions.

Each trial lasted 2.2 s in total and started with the presentation of a central fixation cross for 200 ms, followed by a 900‐ms display presenting the configuration. Next, the (target) dot‐probe was added to the display and presented for another 100 ms near the bottom left or right illusory edge of a given pacman configuration. Finally, a blank screen with a fixation cross was presented again for 1,000 ms. On a given trial, observers were instructed to fixate the central fixation cross. The relatively short duration of the target (100 ms) ensured that observers could not make eye movements toward it. An example trial sequence is shown in Figure 1c. Before the experiment, every participant was acquainted with the tasks. To this end, we used a practice session of 128 trials, which was performed outside the scanner.

In addition, the experiment systematically varied three factors: Task (luminance discrimination, spatial localization), Configuration (Kanizsa, Baseline), and Side (bottom left, bottom right), with all possible factorial combinations presented in random order across blocks. Within a given block, trials with the inside/outside location and light/dark luminance of the target dot were equally frequent but presented in random order across trials.

### 2.4 fMRI measurement

#### 2.4.1 Data acquisition

Functional imaging data were acquired using a 3‐T TRIO MRI system (Siemens, Erlangen, Germany) and T2*‐weighted EPI sequences (repetition time = 2.2 s and echo time = 30 ms). For the experiment, a total of 874 volumes of 36 axial slices were acquired using an interleaved slice mode (thickness = 3 mm, distance factor = 10%, field of view = 200 mm, 64 × 64 matrix, in‐plane voxel size = 3.1 × 3.1 mm^2^).

#### 2.4.2 Data preprocessing

The fMRI data were analyzed using the statistical parametric mapping software SPM12 (Wellcome Department of Imaging Neuroscience, London; http://fil.ion.ucl.ac.uk/spm/software/spm12). As the first five images were acquired before the MR signal had reached its steady state, they were excluded from analysis. To remove sources of noise and artifact, data were preprocessed. Inhomogeneities in the magnetic field were corrected using the fieldmap toolbox (Cusack & Papadakis, 2002). Images were then spatially realigned to correct for interscan movement. Next, the mean EPI image for each participant was computed and spatially normalized to the standard EPI template provided by the Montreal Neurological Institute (MNI) using the “unified segmentation” function in SPM12. The data were then smoothed using a Gaussian kernel of 8 mm full width at half maximum.

### 2.5 Data analysis

#### 2.5.1 Behavioral data analysis

The data from the left and right target‐presentation quadrants were collapsed for the behavioral data analysis. The accuracy and reaction time (RT) data were analyzed using a repeated measures ANOVA with the within‐subject factors Task (luminance, localization) and Configuration (Kanizsa, Baseline).^2^ Trials with very fast responses (RTs < 200 ms) were excluded from the analyses. Error trials were additionally removed before the RT analysis.

#### 2.5.2 Functional analysis: BOLD amplitude—Main experiment

Eight onset regressors were defined, which corresponded to the eight different experimental conditions (2 tasks × 2 configurations × 2 sides). The hemodynamic response was modeled using a canonical hemodynamic response function and its time derivative. Error trials (incorrect/missing responses and trials with RTs faster than 200 ms) were modeled separately. Linear and quadratic effects of the six head movement parameters were included in the design matrix as additional regressors.

To specify the first‐level contrasts, each experimental regressor was compared with the implicit baseline. The resulting contrast images were then subjected to a second level, flexible factorial design with the experimental conditions as within‐subject factors and participants as a random factor, using a random‐effects (mixed‐effects) analysis. We focused on the analysis of the effects of configuration and their interaction with task and hemifield, using planned t‐contrasts. Moreover, to characterize the functional network in the present study, we tested for a positive effect of the hemodynamic response function regressor across all eight conditions in relation to the implicit baseline. All contrasts were thresholded at *p* < .05, with the familywise error (FWE) whole‐brain corrected at the cluster level (with the cluster defining voxel‐level cut‐off set to *p* < .001).

#### 2.5.3 Region of interest definition

Based on our previous findings (Chen et al., 2020), LOC and early visual cortex (V1/V2) were included as possible brain regions for the connectivity models. Selection of the ROIs within each individual was based on a combination of anatomical definitions and group random‐effects analyses testing for differences in BOLD amplitude (see Section 3). The analysis of illusory‐figure effects (Kanizsa > Baseline) revealed significantly higher BOLD amplitudes in the bilateral LOC (right group maxima: 30, −82, 12; left group maxima: −32, −86, 6). The early visual cortex that served as visual input area (right group maxima: 18, −98, 10; left group maxima: −20, −96, 8) for the DCM analysis was identified by comparing all conditions involving visual stimulation against the implicit baseline. The ROI center for each participant was determined by the local maximum (*p* < .05, uncorrected) closest to the peak coordinates from the corresponding group random‐effects analysis within the LOC and V1/V2 masks, as provided by the probabilistic atlas of Wang, Mruczek, Arcaro, and Kastner (2015). Each ROI was a spherical volume with a radius of 3 mm (consisting of 14–19 voxels; see Plewan et al., 2012, for a comparable approach). Mean‐adjusted data (i.e., the first eigenvariate of the time series) from each participant were extracted from all voxels within each ROI.

#### 2.5.4 Dynamic causal modeling

Effective connectivity within a network of brain regions was tested employing DCM (Friston et al., 2003) as implemented in SPM12 (v7771). The following equation expresses the neuronal model that permits to evaluate the changes in neuronal states over time:

$$\dot{z}=\mathrm{Az}+\sum_{j=1}^{m}u_{j}B_{j}z+\mathrm{Cu}$$

In this equation, *z* is the derivative of the hidden neural state for each region, and *u* represents the experimental inputs (Friston et al., 2003). Matrix A stands for the intrinsic coupling between nodes; matrix B represents the modulatory effect of specific inputs on the connectivity between nodes; matrix C encodes the direct effect of a driving input on the hidden neural states. Finally, the posterior distributions of these parameters inform about the impact that different mechanisms have on the dynamics of the model.

Three sets of parameters describe the DCM models: the direct influences of the external input or stimuli on regional activity (i.e., the driving input); the strength of the intrinsic (fixed) connections between two regions in the absence of modulating experimental effects; and the changes in the intrinsic connectivity between regions induced by the experimental design, that is, the modulatory effects of experimental factors. DCM models were specified for each participant individually by setting up a design matrix representing the conditions of interest.^3^ We combined the left and right target sides as no interaction was found between illusion and target hemifield (see below). There were five regressors: one to represent the visual input plus four to model the experimental effects of configuration (Kanizsa, baseline) and task (luminance discrimination, spatial localization). The visual stimulation was then used as the driving input to the model, while Kanizsa and baseline configurations in the discrimination and localization tasks were used to modulate the extrinsic (between‐node) connectivity.

**FIGURE 2:**
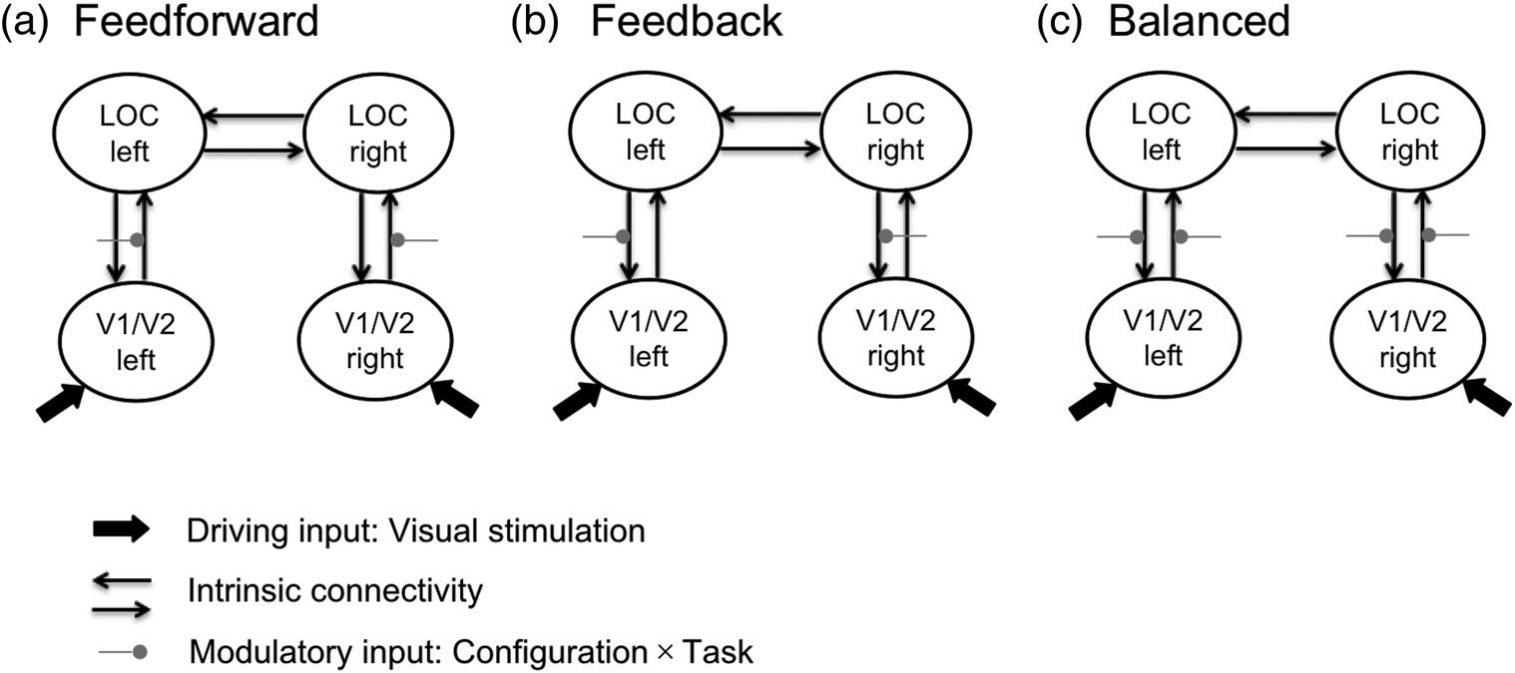
Illustration of the model space as used for the Bayesian model selection (a: feedforward; b: feedback; c: Balanced). The solid black lines depict intrinsic connections between the different ROIs, with the arrows denoting the direction of the respective connections. The gray lines with dots depict the connections that are modulated by a specific experimental condition: Configuration (Kanizsa, Baseline) and/or Task (luminance discrimination, spatial localization). Fat solid arrows denote the driving input region

To determine the model that could best explain the observed responses in LOC and V1/V2, we performed a Bayesian model‐selection (BMS) analysis with a random‐effects (RFX) approach. RFX employs a Gibbs sampling method (Penny et al., 2010; Stephan, Penny, Daunizeau, Moran, & Friston, 2009), taking into account the possibility that different participants might use different models. BMS chooses the winning model based on the exceedance probability and protected exceedance probability, reflecting the likelihood of the model representing the observed data (Penny et al., 2010; Stephan et al., 2010). Figure 2 depicts the model space with three different model variants. In all three models, the fixed, intrinsic connections comprised of reciprocal links between (left/right) V1/V2 and (left/right) LOC, respectively, as well as a reciprocal link between left and right LOC. These connections were taken as core modules for all models tested (Felleman & Van Essen, 1991; Lamme & Roelfsema, 2000; Stephan, Marshall, Penny, Friston, & Fink, 2007). Within each of the three models tested, we additionally varied the modulatory connections between bilateral V1/V2 and LOC as induced by the four experimental (Configuration × Task) conditions: (a) a feedforward model with modulatory connections from V1/V2 to LOC; (b) a feedback model with recurrent connections from LOC to V1/V2; and (c) a balanced model with bidirectional connections between V1/V2 and LOC. Participant‐specific estimates for the parameters of interest in the winning model were then subjected to two‐sided, one‐sample t‐tests to test their differences from zero. In addition, a repeated‐measures ANOVA was computed to test for systematic differences between the experimental factors Configuration and Task. All comparisons used Bonferroni corrections to control for multiple comparisons (at *p* < .05).

The purpose of the DCM model comparison in the current context was to contribute to the debate about the network connectivity underlying perceptual completion processes. Previous studies have proposed either a pure feedforward, bottom‐up process (Ffytche & Zeki, 1996; Grosof et al., 1993; Leventhal et al., 1998; Sheth et al., 1996) or an interactive model that reflects a recurrent network (Lee & Nguyen, 2001; Stanley & Rubin, 2003). If LOC is responding later to the presence of the Kanizsa figure than V1/V2, one would expect to find a forward modulation from V1/V2 to LOC (Figure 2a). In contrast, if LOC initiates a completion signal, the model that best fits the data should include an increase in effective connectivity backward from LOC to V1/V2 in response to a Kanizsa figure (Figure 2b). The third alternative would be a model in which the modulation occurs in both directions, that is, revealing bi‐directional signaling (see Figure 2c). In addition to these analyses, we also report results from a complementary DCM‐parametrical empirical Bayes (PEB) approach in the supplemental materials section (SI).

## 3 RESULTS

### 3.1 Behavioral data

**FIGURE 3:**
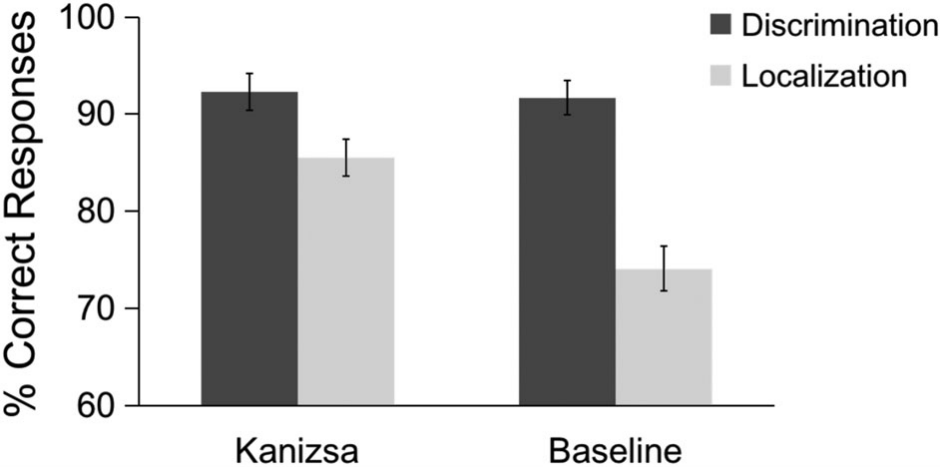
Mean percentage of correct responses for the luminance discrimination and spatial localization tasks (dark and light gray bars, respectively) plotted separately for the Kanizsa and Baseline configurations. Error bars denote 95% (within‐subject) confidence intervals

The mean accuracies across participants are depicted in Figure 3. Participants performed significantly better in the luminance discrimination task (*M* = 92%) compared to the spatial localization task (*M* = 80%), *F* (1, 19) = 75.61, *p* < .0001, *ŋ*_*p*_^2^ = .80. There was also a main effect of Configuration, *F* (1, 19) = 36.15, *p* < .0001, *ŋ*_*p*_^2^ = .66: performance was higher for Kanizsa (*M* = 89%) than for Baseline (*M* = 83%) configurations. The interaction of Task and Configuration was also significant, *F*(1, 19) = 41.77, *p* < .0001, *ŋ*_*p*_^2^ = .69. Follow‐up post hoc comparisons revealed that, while there was no significant difference between configurations in the luminance discrimination task (92.3% and 91.7% for Kanizsa and Baseline configurations, respectively; *p* = .57), there was a significant modulation of performance in the spatial localization task, with higher accuracy for Kanizsa (86%) versus Baseline (74%; *p* < .0001) configurations.

Next, for the analysis of the mean RTs, a comparable ANOVA revealed a marginally significant main effect of Configuration, *F*(1, 19) = 3.73, *p* = .07, *ŋ*_*p*_^2^ = .16: RTs were 8 ms faster in response to the Kanizsa diamond versus the Baseline configuration. The effect of Task was not significant (*p =* .24, *ŋ*_*p*_^2^ = .07). However, there was a significant Task × Configuration interaction, *F*(1, 19) = 5.67, *p* = .028, *ŋ*_*p*_^2^ = .23, which was due to RTs being comparable between the two configurations in the luminance discrimination task (529 ms and 528 ms for Kanizsa and Baseline, respectively; *p* = .93), but faster in response to Kanizsa (533 ms) than to Baseline (550 ms; *p* = .03) configurations in the spatial localization task. Together, this pattern shows that an object benefit in behavioral measures was evident only in the localization task, in which the spatial configuration was task relevant. Thus, performance depended on both task and configuration variations.

### 3.2 Whole‐brain data

**FIGURE 4:**
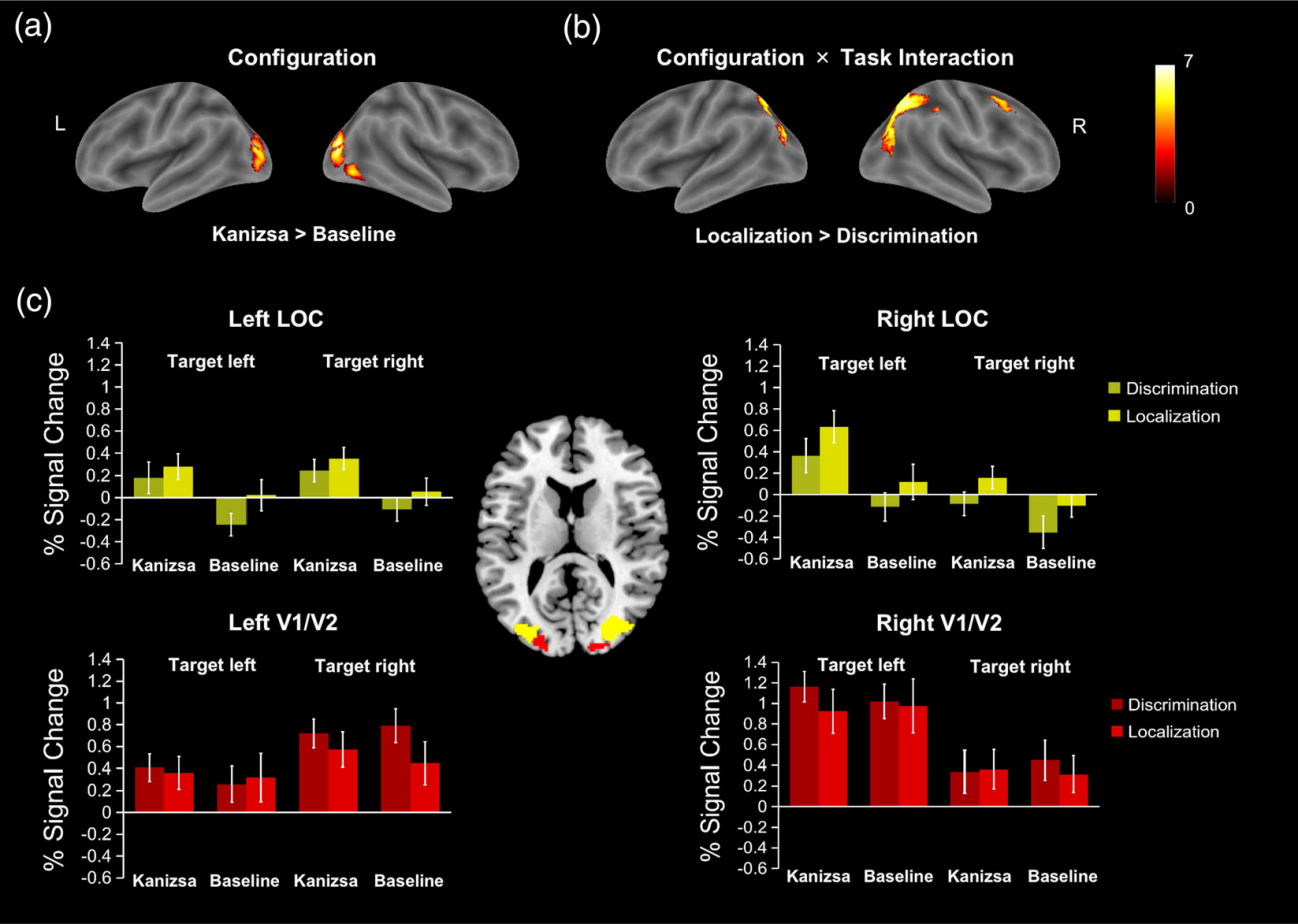
Surface rendering of the functional magnetic resonance imaging (fMRI) activations as obtained in the whole‐brain analysis: panel a depicts the activations related to the emergence of an illusory figure, while panel b illustrates the differential effect of the illusory figure (Kanizsa > Baseline) in the spatial localization and luminance discrimination tasks. All contrasts were thresholded at *p* < .05 familywise error, whole‐brain corrected at the cluster‐level (with a cluster‐defining voxel‐level cut‐off of *p* < .001). c. Neural activity modulated by experimental conditions (task × configuration × target side) in the four ROIs, with BOLD responses in V1/V2 (the red area in the middle panel) and LOC (the yellow area in the middle panel) in bilateral hemispheres. Error bars denote the standard error of the mean (SEM)

Comparisons of all conditions that involved a visual stimulation with the implicit baseline revealed activations in the putamen, cerebellum, early visual areas (mainly in V2), middle frontal gyrus, and precentral gyrus (Table 1). Neural effects associated with the representation of the illusory (diamond) object were additionally examined by contrasting configurations that led to the emergence of an illusory figure with the ungrouped baseline configuration (Table 1; Figure 4a). Activations positively associated with the generation of a complete illusory figure (Kanizsa) were detected bilaterally in LOC, extending from the inferior occipital gyrus (IOG) to the middle occipital gyrus (MOG) and into the fusiform gyrus. To test the effects induced by the different tasks on the coding of the illusory figure, we further compared the activation pattern as induced by the complete illusory figure (Kanizsa > Baseline) between the luminance discrimination and spatial localization task. The interaction term was analyzed for both effect directions, with either a stronger (or, conversely, a weaker) activation induced by the illusory figure in the spatial localization task compared to the luminance discrimination task. The first comparison (localization > discrimination) revealed clusters of activation covering the bilateral superior parietal lobule (SPL) and MOG as well as at the posterior end of the right middle frontal cortex (Table 1; Figure 4b). No significant results were obtained for the interaction term that tested task effects in the opposite direction (localization < discrimination). Finally, we also tested the interaction between the illusory figure (Kanizsa > Baseline) and the target (dot) hemifield, which, however, showed no reliable effect. Together, these results show that object completion was associated with LOC activations, while grouping effects that were specifically associated with the spatial localization task were related to activity in parietal, occipital, and frontal areas.

**TABLE 1:** List of activations associated with visual stimulation, the emergence of an illusory Kanizsa figure, and the respective interaction of Configuration with Task and Hemifield

Region label	Cluster size	MNI coordinates			*T*
All trials > implicit baseline					
L Putamen	1,273	−24	−4	10	7.11
R Putamen	667	24	0	10	6.44
R Cerebellum	586	8	−74	−20	6.53
	515	42	−58	−28	7.40
L Cerebellum	495	−40	−62	−30	6.41
R Cuneus/V2	82	18	−98	10	5.87^a^
L Middle Occipital Gyrus/V2	163	−20	−96	8	4.55^a^
R Middle Frontal Gyrus	106	48	4	56	5.14^a^
L Precentral Gyrus	53	−46	−4	58	4.83^a^
Configuration effect (Kanizsa > Baseline)					
R Middle occipital gyrus/LOC	1,018	30	−82	12	7.21
R Inferior occipital gyrus/LOC		40	−76	−4	5.62
R Fusiform gyrus		32	−60	−6	3.72
L Middle occipital gyrus/LOC	556	−32	−86	6	5.91
L Inferior occipital gyrus/LOC		−34	−78	−4	4.34
Configuration (Kanizsa > Baseline) × Task interaction					
Localization > Discrimination					
R Superior parietal lobule	2,692	20	−64	62	5.19
R Middle occipital gyrus		38	−76	28	4.47
L Superior parietal lobule	764	−24	−66	50	4.78
L Middle occipital gyrus		−38	−82	22	4.19
R Superior frontal gyrus	500	18	14	70	4.02
R Middle frontal gyrus		42	30	40	3.62
Discrimination > Localization					
n.s.					
Configuration (Kanizsa > Baseline) × Hemifield interaction					
n.s.					

*Note*: All activations significant at *p* < .001, with FWE cluster correction at *p* < .05.

^a^ At *p* < .001 uncorrected and in a small‐volume correction at *p* < .05 FWE corrected. The small volume consisted of a sphere with a diameter of 10 mm at the positions defined by the respective a priori hypothesis.

A subsequent comparison then included bilateral LOC (right group maxima: 30, −82, 12; left group maxima: −32, −86, 6) and early visual cortex (V1/V2; right group maxima: 18, −98, 10; left group maxima: −20, −96, 8) as ROIs, based on the group random‐effects analyses (see above and Section 2). The beta values representing BOLD amplitudes were extracted from the voxels within the different ROIs and were then compared for each ROI using a repeated‐measures ANOVA with the factors Task, Configuration, and Target side. The BOLD signals of the left and right V1/V2 (Figure 4c, red) revealed only a main effect of target side for right V1/V2, *F*(1, 19) = 5.27, *p =* .03, *ŋ*_*p*_^2^ = .22, with higher signals for left‐ than right‐side targets. No other effects were significant (all *p*s > .25, *ŋ*_*p*_^2^ < .07). Next, the BOLD signals for left and right LOC (Figure 4c, yellow) were examined. For the left LOC, there was a main effect of configuration, *F*(1, 19) = 11.74, *p =* .003, *ŋ*_*p*_^2^ = .38, with higher signals for the Kanizsa than the Baseline configuration (irrespective of the target side and task). For the right LOC, all main effects were significant, task: *F*(1, 19) = 4.52, *p =* .047, *ŋ*_*p*_^2^ = .19; configuration: *F*(1, 19) = 14.77, *p =* .001, *ŋ*_*p*_^2^ = .44; target side: *F*(1, 19) = 4.42, *p =* .049, *ŋ*_*p*_^2^ = .19. Signals were higher for the localization task versus the discrimination task; they were also higher for the Kanizsa figure than for the Baseline configuration, and for left‐side as compared to right‐side targets. All other effects were nonsignificant, *p* > .12, *ŋ*_*p*_^2^ < .12. This pattern of results thus supports previous findings, which demonstrated object completion effects to be typically associated with LOC activations (e.g., Kourtzi & Kanwisher, 2001; Mendola et al., 1999; Stanley & Rubin, 2003).

### 3.3 Dynamic causal modeling

The primary goal of this study was to understand the connectivity dynamics between structures of the LOC and V1/V2 in the coding of illusory figures (when the emerging object is vs. is not task relevant). Accordingly, we performed a DCM analysis on time series extracted from representative ROIs as identified in the whole‐brain analyses described above (see also Figure 4c). For the DCM analysis, three models were constructed to test how the Kanizsa figure was processed. The models all assumed reciprocal intrinsic connections with V1/V2 serving as the driving input, but they differed in terms of their modulatory parameters, that is, how the configuration modulated the connectivity between regions. In the feedforward model, V1/V2 would initiate the completion and propagate corresponding signals to LOC (Figure 2a); in the feedback model (Figure 2b), in contrast, V1/V2 becomes involved only in later‐stage processing of the completed configuration—receiving feedback signals from LOC. Finally, in a third, alternative model, the signals modulated by the configuration were reciprocal, with bidirectional connections between V1/V2 and LOC (Figure 2c).

**FIGURE 5:**
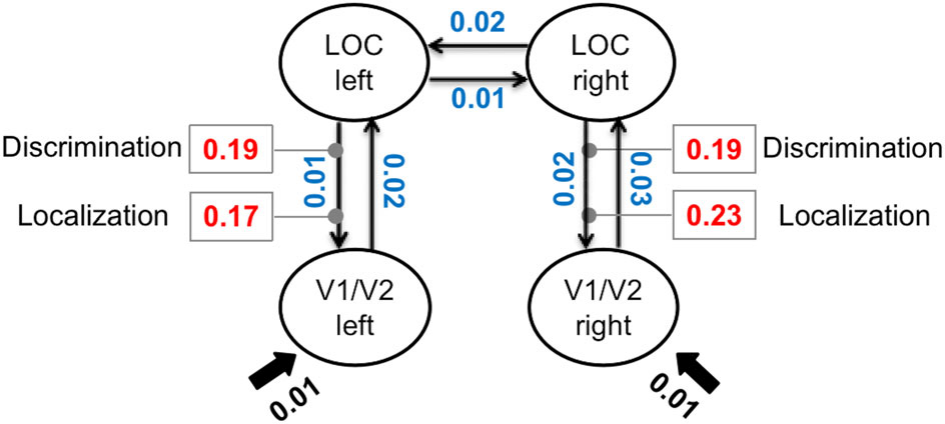
Parameter values for the winning “feedback” model in response to the Kanizsa figure in the two task conditions resulting from the dynamic causal modeling (DCM) analysis. The numbers next to the connections represent average parameter estimates from the group (note that all effects displayed were significant). The lines with arrows depict intrinsic connections between the different ROIs, with the mean parameter values shown in blue. The gray lines with dots represent a connection modulated by the specific experimental task given a stimulation with the Kanizsa figure. The mean modulatory parameter values are depicted in the boxes (shown in red). Fat solid arrows represent the driving input, with the mean parameter values shown in black

Bayesian model comparisons were applied to select the model with the highest exceedance probability (xp) and protected exceedance probability (pxp). Comparisons of the three models revealed the feedback model to be clearly superior to the other two models (feedback model: xp = 0.97, pxp = 0.81; feedforward model: xp = 0.02, pxp = 0.1; balanced model: xp = 0.01, pxp = 0.09). In the next step, the connectivity parameters, that is, the intrinsic connections and the condition‐dependent modulations of the winning feedback model were entered into a second‐level analysis, using two‐tailed, one‐sample t‐tests to compare individual connection strengths against zero (at *p* < .05, Bonferroni corrected for multiple comparisons). The results are summarized in Table 2. And Figure 5 presents the mean significant parameter estimates of this feedback model for the Kanizsa figure configuration in the two task conditions.

**TABLE 2:** Mean parameter estimates for the winning model (with feedback modulation)

Connection/parameter	Mean	*SD*	*t*	*p*
Intrinsic connections				
Left V1/V2 → LOC	0.02	0.03	2.26	.036*
Right V1/V2 → LOC	0.03	0.07	2.20	.04*
Left LOC → V1/V2	0.01	0.01	4.84	.0001***
Right LOC → V1/V2	0.02	0.03	2.46	.024*
Left LOC → Right LOC	0.01	0.01	3.74	.001***
Right LOC → Left LOC	0.02	0.03	2.62	.017**
Driving input				
Left V1/V2	0.01	0.02	2.26	.036*
Right V1/V2	0.01	0.03	2.41	.026*
Modulation by the Kanizsa figure				
Left LOC → V1/V2 by Discrimination	0.19	0.25	3.38	.003**
Right LOC → V1/V2 by Discrimination	0.19	0.26	3.19	.005**
Left LOC → V1/V2 by Localization	0.17	0.27	2.72	.014*
Right LOC → V1/V2 by Localization	0.23	0.29	3.52	.002**
Modulation by the Baseline configuration				
Left LOC → V1/V2 by Discrimination	0.01	0.35	0.15	.88
Right LOC → V1/V2 by Discrimination	0.03	0.37	0.39	.70
Left LOC → V1/V2 by Localization	−0.04	0.32	−0.57	.57
Left LOC → V1/V2 by Localization	0.06	0.32	0.80	.43

*Note*: Bonferroni corrected for multiple comparisons; two‐tailed. *N* = 20.

* Significant at *p* < .05.

** Significant at *p* < .01.

*** Significant at *p* < .001.

The driving inputs into bilateral V1/V2 were both significant and positive, left: *t*(19) = 2.26, *p* < .05 (mean: 0.01 Hz); right: *t*(19) = 2.41, *p* < .05 (mean: 0.01 Hz). Moreover, intrinsic connections were also all positive, with significant connections from bilateral V1/V2 to LOC, left: *t*(19) = 2.26, *p* < .05 (mean: 0.02 Hz); right: *t*(19) = 2.20, *p* < .05 (mean: 0.03 Hz); and from bilateral LOC to V1/V2, left: *t*(19) = 4.84, *p* < .001 (mean: 0.01 Hz); right: *t*(19) = 2.46, *p* < .05 (mean: 0.02 Hz). In addition, the reciprocal connections between right LOC and left LOC were significant, left LOC to right LOC: *t*(19) = 3.74, *p* < .001 (mean: 0.01 Hz); right LOC to left LOC: *t*(19) = 2.62, *p* < .05 (mean: 0.02 Hz).

Finally, the modulatory parameters (induced by the experimental variations) on the connections from bilateral LOC to V1/V2 were significant and positive for the Kanizsa figure in both tasks, all *t* > 2.72, *p* < .014; in contrast, the parameter values did not differ reliably from zero for the Baseline configuration, all *t* < .80, *p* > .43. To further investigate the pattern of the modulatory effects, a repeated‐measures ANOVA with the factors Configuration (Kanizsa, Baseline), Task (luminance discrimination, spatial localization), and Hemisphere (left, right) was computed. The results revealed a significant main effect of Configuration, *F*(1, 19) = 12.48, *p =* .002, *ŋ*_*p*_^2^ = .40, reflecting a stronger effect for the Kanizsa figure (M = 0.19 Hz) vs. the Baseline configuration (M = 0.02 Hz). However, no main and interaction effects that involved the factors Task or Hemisphere were significant, all *F* < 2.02, *p* > .17, *ŋ*_*p*_^2^ < 0.10. This pattern suggests that the presence of a Kanizsa figure in the stimulus display leads to an increase in the connection strength (providing feedback from LOC to V1/V2), which is independent of the specific task demands and comparable across the two hemispheres. Thus, in both the left and right hemispheres, LOC activity had a causal effect on the activation in V1/V2—supporting a specific role of the early visual cortex in response to the Kanizsa figure at some later processing stages, that is, after feedback signals from LOC reach the earlier visual areas. A complementary DCM‐PEB approach (see supplemental materials) confirmed a significant feedback connection from LOC to V1/V2, which was modulated by the Kanizsa figure in both discrimination and localization tasks, though this effect appeared to be reliable only in the right hemisphere.

## 4 DISCUSSION

Illusory figures serve as a prominent example to demonstrate the efficiency of perceptual grouping in human vision. In the present study, we used fMRI in combination with DCM to investigate the response profile and the connectivity dynamics between regions that have been implicated in illusory figure processing, namely: bilateral LOC and early visual cortex (with the latter processing the initial stimulus input). In our paradigm, the processing of Kanizsa figures was compared in two tasks that varied in their attentional demands (Chen et al., 2018; Chen et al., 2020). In the spatial localization task, participants localized a dot‐probe as inside versus outside the presented configuration, making the presented grouping task relevant. In the luminance discrimination task, participants judged the brightness of the very same dot‐probe. That is, the object configurations were not directly relevant for successful task performance. The behavioral results replicated previous findings, showing that the completed object facilitated performance in the spatial localization task but not in the luminance discrimination task. Thus, a behavioral object benefit manifests in particular when the spatial organization of the display is relevant for the task at hand.

Our neuroimaging data showed that the appearance of a Kanizsa figure produced reliable activations predominantly in mid‐level visual processing areas, particularly in the bilateral LOC, with stronger object‐specific activations in the right hemisphere. Overall, these findings are consistent with most neuroimaging and electrophysiological studies, which reported illusory figure processing to be associated with LOC activations (Halgren et al., 2003; Kruggel, Herrmann, Wiggins, & von Cramon, 2001; Mendola et al., 1999; Murray et al., 2004; Shpaner et al., 2013; Shpaner, Murray, & Foxe, 2009; Stanley & Rubin, 2003). Moreover, we found an interaction between object completion and the task specification (spatial localization vs. luminance discrimination): the spatial localization task led to a more pronounced object benefit, which was associated with activations in SPL and MOG as well as the posterior end of the right middle frontal cortex. In other words, attending specifically to the object configuration for performing the spatial localization task (in which the configuration was task relevant) was associated with several activations in occipital, parietal, and frontal regions, with more significant activations in the right hemisphere. The opposite contrast (luminance discrimination vs. spatial localization) did not reveal any task‐specific activations associated with discerning the dot‐probe as being light or dark. Previous studies also found that the right superior parietal cortex is involved in spatial localization (e.g., Fink et al., 2000; Fink, Marshall, Weiss, & Zilles, 2001; Plewan et al., 2012; Weidner & Fink, 2007). Our findings might thus reflect the varying degrees with which the representation of the illusory figure has to be taken into account to solve the respective task.

Besides the findings from the whole‐brain analysis—which replicate previous results—the main goal of the experiment was to use DCM to test the effective connectivity between processing nodes implicated in object completion, namely, LOC and early visual cortex. The DCM results favored a model that comprised a significant, positive feedback modulation with connections back‐projecting from LOC to the early visual cortex for the presentation of Kanizsa figures (whereas there was no comparable, significant modulation for ungrouped “baseline” configurations). Of note, the feedback connection modulated by the Kanizsa figure did not differ between the two tasks, despite the clear difference in the amount of attentional engagement toward the illusory figure in the localization versus the discrimination task. In other words, the DCM analysis did not reveal differences in the effective connectivity associated with object grouping as a function of the relevance of grouping to the task at hand. This argues in favor of the completion of Kanizsa figures in V1/V2 and LOC operating essentially independently of the task demands. Instead, task‐specific differences might be implemented at a later stage of processing, for instance, as reflected in interactions between the parietal cortex and LOC (see Plewan et al., 2012).

The specific increase in backward connection strength induced by presentation of Kanizsa figures suggests that completion of the illusory diamond shape has a delayed effect on early visual cortex, with shape‐specific processing being modulated by feedback projections from LOC. This finding is inconsistent with accounts that assume early visual cortex to initiate object completion, that is, as constituting the first stage of the object‐integration process. Instead, the early visual cortex appears to be functionally involved mainly at a later stage of processing the completed object, for instance, when re‐evaluating recurrent signals from a hierarchically higher processing level. This interpretation is consistent with Stanley and Rubin's (2003) account of Kanizsa‐figure completion, according to which completion of illusory objects is mainly driven by recurrent input from LOC to early visual areas V1/V2, with interactions between mid‐level and lower‐tier visual areas in the visual hierarchy engaging in object completion via recurrent processes. Our results are also broadly consistent with recent studies that assessed the timing of initial illusory figure processing using EEG and MEG (Halgren et al., 2003; Kruggel et al., 2001; Murray et al., 2002, 2004; Shpaner et al., 2009, 2013), which reported that the neural response to Kanizsa figures peaks earlier in LOC than in V1/V2.

Moreover, a DCM‐PEB analysis (see Supplemental Materials) also showed that Kanizsa figure in both tasks induced a strong excitatory feedback exerted by right LOC onto right V1/V2 (while not providing reliable evidence for such a pattern in the left hemisphere). This right‐hemispheric lateralization essentially mirrors the overall trends as observed in the main analyses (see above; see also Chen et al., 2020), and might reflect the fact that the PEB approach is stricter due to the quantification of the within‐participants variability of the connectivity parameters (Friston et al., 2003). Indeed, some studies have reported lateralization effects, with illusory figures tending to activate the right hemisphere more than the left (Hirsch et al., 1995; Larsson et al., 1999; Halgren et al., 2003; see also Fink et al., 1996) and that patients with right‐hemispheric, posterior lesions are impaired at perceiving illusory figures, whereas patients with left‐hemisphere lesions exhibit no difference relative to controls (Grabowska, Nowicka, Szymańska, & Szatkowska, 2001; Wasserstein, Zappulla, Rosen, Gerstman, & Rock, 1987).

The importance of (right‐hemispheric) feedback connections for efficient illusory figure completion may in turn be related to the functional architecture of the visual system. For instance, given that the receptive fields of LOC neurons are much larger than those in V1 and V2 (Motter, 2009; Pollen, Przybyszewski, Rubin, & Foote, 2002), LOC may support the integration of local features into a global shape, allowing surfaces to be segmented from the background (Grill‐Spector, 2003; Lamme, 1995; Pasupathy & Connor, 2002; Vuilleumier, Henson, Driver, & Dolan, 2002). Once the integration of local stimulus features is completed, the global shape information would then be transmitted back to the early visual areas V1 and V2 to “work out the details,” such as to strengthen the figure‐ground segregation process and define the contours that demarcate the boundary of the segmented illusory figure (Roelfsema, 2006; Seghier & Vuilleumier, 2006). Early visual areas with small receptive fields appear to be optimal for encoding information with high spatial precision and resolution, thus being able to render local details such as the edges and contours of an object.

### Conclusions

We used fMRI in combination with DCM to investigate the connectivity dynamics between LOC and the early visual cortex. We found a specific activation pattern in LOC in response to Kanizsa figures relative to ungrouped configurations, confirming the previously reported, essential role of LOC in the object‐completion process. Most importantly, we also demonstrate, for the first time (with fMRI‐specific methods), that the significant modulation of effective connectivity in response to the Kanizsa figure was associated with an increase in the coupling strength of feedback signals from LOC to V1/V2, independently of the current task demands. We thus conclude that the neural representation of the illusory figure may be achieved by progressive integration of local features into a global representation of the whole through a feedback pathway from LOC to V1/V2: LOC engages in the extraction of the overall shape of the completed object, while the early visual cortex is subsequently involved in determining the specific local details of the integrated object.

## CONFLICT OF INTEREST

The authors have declared that no competing interests exist.

## Supporting information


**Appendix** **S1:** supporting information

## Data Availability

The original data are available from the first author on request.

## Endnotes

^1^
Note that two additional stimulus configurations, which presented (a) a shape configuration that depicted partial contour and surface completions (Shape), and (b) a configuration that only induced an illusory contour without an associated surface (Contour), were also included in the experiment (see Chen et al., 2020 for a detailed description). However, we excluded these two conditions from the present analyses because the current study primarily aimed to present differences in effective connectivity for the two most distinct types of stimuli: a fully grouped illusory figure as opposed to a baseline (ungrouped) configuration without *any* emerging shape.

^2^
Additional analyses on the accuracies, RTs, and DCM parameters (see below) were performed with an additional factor, “age” as a covariate. However, these analyses did not change the pattern of results, and there was no interaction between the covariate age and the experimental factors (all *p*'s > .25). This indicates that our reported effects were little influenced by variability in age.

^3^
In order to perform the DCM analysis, data preprocessing was conducted again with a correction for differences in slice‐timing acquisition for each whole‐brain volume additionally after the realignment.
