# Supplementary material for: Feedback from lateral occipital cortex to V1/V2 triggers object completion: Evidence from functional magnetic resonance imaging and dynamic causal modeling
Source: Hum Brain Mapp. 2021 Aug 21;42(17):5581–94. doi: 10.1002/hbm.25637 (PMC8559483; doi:10.1002/hbm.25637)
Supplement: Supplementary file 1 — Appendix S1: supporting information [file HBM-42-5581-s001.docx]

Supplementary Information

**Feedback from LOC to V1/V2 triggers object completion – evidence from fMRI and dynamic causal modeling**

Siyi Chen^1^, Ralph Weidner^2^, Hang Zeng^3^, Gereon R. Fink^2,4^,

Hermann J. Müller^1^, Markus Conci^1^

^1^Department of Psychology, Ludwig-Maximilians-Universität München, 80802 München, Germany

^2^Cognitive Neuroscience, Institute of Neuroscience and Medicine (INM-3),

Research Center Jülich, 52428 Jülich, Germany

^3^Center for Educational Science and Technology, Beijing Normal University at Zhuhai, 519087 Zhuhai, China

^4^Department of Neurology, University Hospital Cologne, Cologne University, 50937 Cologne, Germany

Running title: Feedback triggers object completion

DCM-Parametrical Empirical Bayes (PEB) analyses

To compare the changes in connectivity caused by the different experimental conditions (Configuration × Task), we used a DCM-PEB approach complementary to the “regular” DCM modeling approach as presented in the main manuscript. Parametrical Empirical Bayes (PEB) (Friston et al., 2015, 2016) is a hierarchical Bayesian model that combines both non-linear (at the first level) and linear (at second level) analyses. The main advantage of using PEB is to assess commonalities and differences among subjects in terms of effective connectivity at the group level in order to take into account the variability of the individual connection strength (Zeidman et al., 2019a, 2019b). This variance of estimated uncertainty regarding the connection strengths is typically not considered in classical statistical tests.

In the current DCM-PEB analysis, we specified two ROIs in the bilateral V1/V2 regions and two ROIs in the bilateral LOC. Individual ROIs were created by using the eigenvariate extracted from a sphere with a radius of 6 mm. The sphere’s center was located at the nearest local maximum within 10 mm from the corresponding ROI centers as listed in Methods section in the main manuscript. The mean distance was about 28 mm (SD = 7 mm) between the left ROIs and 26 mm (SD = 8 mm) between the right ROIs.

First, a full DCM was specified and estimated for each participant using SPM12 (cf., SPM procedure: spm_dcm_fit). The full model allows all connectivity parameters in all directions to be freely estimated. We specified a bilinear deterministic DCM without centering around the mean (Friston et al., 2003), which included (a) all forward and backward fixed connections between the ROIs (matrix A), (b) all the modulatory connections or parameters that reflect conditional changes due to each experimental condition (matrix B) and (c) direct input parameters that reflect the input driving the activity in V1/V2 in all experimental and control conditions (matrix C). That is, the driving input in matrix C consists of one vector with all the onsets of all experimental and control conditions combined as one input, and the modulatory connections in second matrix B are specified only for the experimental conditions. Second, a Parametric Empirical Bayes (PEB) model was constructed for the whole group of participants across all parameters (cf., SPM procedure:spm_dcm_peb). This enabled us to estimate the effective connectivity averaged across all participants (cf., group average), taking into account the within-participants variability on the connectivity parameters. Since we were primarily interested in the group means, other between-subjects effects were not modeled. Consequently, we used a between-subject design matrix X = [1...1]^T^. The PEB approach automatically excluded those connectivity parameters from the group-level PEB that did not contribute to the model evidence, using Bayesian model reduction (BMR; cf. SPM procedure:spm_dcm_peb_bmc). BMR has the advantage that any reduced model at the group level can be estimated efficiently without having to re-estimate the reduced models at the single-participant level. Specifically, a greedy search iteratively compared the full model with 256 models where one or more connections with least evidence are pruned out and thus switched off, whereas the parameters with the most evidence are kept stable so that the most relevant nested models from the full PEB model are tested (Friston & Penny, 2011; Friston et al., 2016). Next, Bayesian model averaging (BMA) of the parameters across models were applied and used for group-inferences (e.g., Penny et al., 2006). We used a threshold based on free energy, taking into account the covariance of parameters, and considered connectivity parameters as significant when their posterior probability > 0.95 (based on model comparisons with and without each parameter).

**Results**

The intrinsic coupling between nodes (as indexed by matrix A) is illustrated in Figure S1, with the connection strengths depicted by the numbers in blue. Our results showed that the right V1/V2 and right LOC had a reciprocal positive influence. In addition, the modulatory effect of the Kanizsa figure (as reflected by the B matrices), in both the luminance discrimination and localization tasks was revealed by a strong excitatory feedback exerted by right LOC to right V1/V2. No significant modulatory effects were observed for the Baseline configuration. In addition, none of the driving inputs to V1/V2 reached significance. Thus, these results support an account of object completion in terms of feedback processing (thus, essentially confirming the results from the “regular” DCM analysis as presented in the main analysis), further demonstrating that this completion effect is particularly strong in the right hemisphere (while there were no significant effects in the left hemisphere).


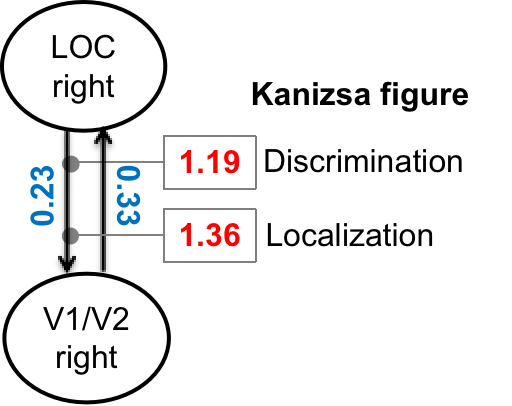


**Figure S1**. DCM-PEB results. All connections denote a posterior probability > .95. The connectivity estimates correspond to rate constants and are expressed in units of 1/s (Hz). The lines with arrows depict intrinsic connections between the different ROIs, with the mean parameter values shown in blue. The grey lines with dots represent a connection modulated by the Kanizsa figure in the discrimination and localization tasks. The mean modulatory parameter values are depicted in the boxes (shown in red).

**References**

Friston, K. J., Harrison, L., & Penny, W. (2003). Dynamic causal modeling. *Neuroimage, 19*(4), 1273–1302. https://doi.org/10.1016/S1053-8119(03)00202-7.

Friston, K. J., Litvak, V., Oswal, A., Razi, A., Stephan, K.E., van Wijk, B. C. M., Ziegler, G., & Zeidman, P. (2016). Bayesian model reduction and empirical Bayes for group (DCM) studies. *Neuroimage, 128*, 413–431. https://doi.org/10.1016/j.neuroimage.2015.11.015

Friston, K. J. & Penny, W. (2011). Post hoc Bayesian model selection. *Neuroimage, 56*, 2089–2099. https://doi.org/10.1016/j.neuroimage.2011.03.062

Friston, K. J., Zeidman, P., & Litvak, V. (2015). Empirical Bayes for DCM: A Group Inversion Scheme. *Frontiers in Systems Neuroscience*, *9*, 164. https://doi.org/10.3389/fnsys.2015.00164

Penny, W., Mattout, J., & Trujillo-Barreto, N. (2006). Bayesian model selection and averaging. *Statistical Parametric Mapping: The Analysis of Functional Brain Images. London: Elsevier*.

Zeidman, P., Jafarian, A., Corbin, N., Seghier, M. L., Razi, A., Price, C. J., & Friston, K. J. (2019a). A guide to group effective connectivity analysis, part 1: First level analysis with DCM for fMRI. *NeuroImage*, *200*, 174–190. https://doi.org/10.1016/j.neuroimage.2019.06.031

Zeidman, P., Jafarian, A., Seghier, M. L., Litvak, V., Cagnan, H., Price, C. J., & Friston, K. J. (2019b). A guide to group effective connectivity analysis, part 2: Second level analysis with PEB. *NeuroImage*, *200*, 12–25. https://doi.org/10.1016/j.neuroimage.2019.06.032
